# Quantitative PCR analysis of salivary pathogen burden in periodontitis

**DOI:** 10.3389/fcimb.2015.00069

**Published:** 2015-10-01

**Authors:** Aino Salminen, K. A. Elisa Kopra, Kati Hyvärinen, Susanna Paju, Päivi Mäntylä, Kåre Buhlin, Markku S. Nieminen, Juha Sinisalo, Pirkko J. Pussinen

**Affiliations:** ^1^Oral and Maxillofacial Diseases, University of Helsinki and Helsinki University HospitalHelsinki, Finland; ^2^Division of Periodontology, Department of Dental Medicine, Karolinska InstitutetHuddinge, Sweden; ^3^Department of Cardiology, Heart and Lung Center, Department of Medicine, Helsinki University HospitalHelsinki, Finland

**Keywords:** cumulative approach, oral pathogen, pathogen burden, periodontitis, quantitative PCR, saliva, salivary diagnostics

## Abstract

Our aim was to investigate the value of salivary concentrations of four major periodontal pathogens and their combination in diagnostics of periodontitis. The Parogene study included 462 dentate subjects (mean age 62.9 ± 9.2 years) with coronary artery disease (CAD) diagnosis who underwent an extensive clinical and radiographic oral examination. Salivary levels of four major periodontal bacteria were measured by quantitative real-time PCR (qPCR). Median salivary concentrations of *Porphyromonas gingivalis, Tannerella forsythia*, and *Prevotella intermedia*, as well as the sum of the concentrations of the four bacteria, were higher in subjects with moderate to severe periodontitis compared to subjects with no to mild periodontitis. Median salivary *Aggregatibacter actinomycetemcomitans* concentrations did not differ significantly between the subjects with no to mild periodontitis and subjects with moderate to severe periodontitis. In logistic regression analysis adjusted for age, gender, diabetes, and the number of teeth and implants, high salivary concentrations of *P. gingivalis, T. forsythia*, and *P. intermedia* were significantly associated with moderate to severe periodontitis. When looking at different clinical and radiographic parameters of periodontitis, high concentrations of *P. gingivalis* and *T. forsythia* were significantly associated with the number of 4–5 mm periodontal pockets, ≥6 mm pockets, and alveolar bone loss (ABL). High level of *T. forsythia* was associated also with bleeding on probing (BOP). The combination of the four bacteria, i.e., the bacterial burden index, was associated with moderate to severe periodontitis with an odds ratio (OR) of 2.40 (95% CI 1.39–4.13). When *A. actinomycetemcomitans* was excluded from the combination of the bacteria, the OR was improved to 2.61 (95% CI 1.51–4.52). The highest OR 3.59 (95% CI 1.94–6.63) was achieved when *P. intermedia* was further excluded from the combination and only the levels of *P. gingivalis* and *T. forsythia* were used. Salivary diagnostics of periodontitis has potential especially in large-scale population studies and health promotion. The cumulative strategy appears to be useful in the analysis of salivary bacteria as markers of periodontitis.

## Introduction

Periodontitis is a multifactorial and multibacterial disease of the supporting tissues of teeth initiated by disturbances in the subgingival biofilm and host homeostasis. The symptoms of the disease involve gingival swelling and bleeding, formation of deepened periodontal pockets, and inflammatory destruction of periodontal ligament and alveolar bone. Finally, untreated periodontitis may lead to the loss of teeth.

The tissue destruction results from the host defense response against bacterial challenge. The progression of periodontitis is characterized by an increase in subgingival bacterial load and by the transformation of the dominance of Gram-positive bacteria to a majority of Gram-negative bacteria. The bacteria associated with periodontitis have been classified into five major microbial color-coded complexes, since these bacteria are repeatedly found together in periodontitis. The “red complex” periodontopathogens, including *Porphyromonas gingivalis, Tannerella forsythia*, and *Treponema denticola*, have a particularly strong association with periodontitis-related clinical parameters such as pocket probing depth (PPD) and bleeding on probing (BOP) (Socransky et al., 1998). The “orange complex” involves typical periodontopathogens related to PPD, e.g., *Prevotella intermedia* (Socransky et al., 1998). In addition, *Aggregatibacter actinomycetemcomitans* is among the bacteria involved in the pathology of periodontitis (Genco, 1996; Henderson et al., 2010; Könönen and Müller, 2014).

There are numerous methods to detect and quantify periodontal bacteria, e.g., microbial cultivation, species-specific DNA probes, or conventional end-point PCR. All these methods have limitations to measure the concentrations of specific bacteria accurately. Most of the previous reports on the number of periodontal bacteria use subgingival plaque, not saliva, as sample material. The presence of salivary bacteria, as well as the number of bacterial species in saliva, have been associated with periodontitis and periodontitis-related variables in several previous studies (Umeda et al., 1998; Könönen et al., 2007; Paju et al., 2009). However, the number of studies investigating salivary bacterial concentrations determined by qPCR and especially the combinations of multiple bacteria is limited (Hyvärinen et al., 2009; Saygun et al., 2011). Moreover, the number of subjects in these studies has been relatively small.

Saliva is a promising diagnostic fluid and it has been widely analyzed for biomarkers of health and disease over the past decade (Giannobile et al., 2011). The benefit of saliva samples over subgingival bacterial samples is that saliva is inexpensively and easily collected (Zhang et al., 2009). Saliva samples can be taken by non-dental healthcare professionals or even by the patients themselves. The relative levels of periodontal pathogens seem to be similar in whole saliva or mouthwash compared to periodontal lesions (Umeda et al., 1998; Boutaga et al., 2007; Haririan et al., 2014). Moreover, saliva reflects the overall conditions in mouth; in addition to tooth surfaces and periodontal pockets, periodontal pathogens can also be found on tongue and mucosa.

In addition to periodontitis, various systemic conditions, and behavioral factors such as smoking may affect salivary biomarkers and bacterial composition (Mager et al., 2003). For example, our previous study showed that high salivary concentrations of *A. actinomycetemcomitans* were associated with increased risk for coronary artery disease (CAD) (Hyvärinen et al., 2012).

In this study, we investigated the salivary levels of four important periodontal pathogens, *P. gingivalis, T. forsythia, P. intermedia*, and *A. actinomycetemcomitans*, in 462 individuals who underwent a detailed periodontal examination. Overall, the study population represents systemically compromised middle-aged and older subjects who are known to frequently suffer from periodontal disease. Our aim was to study if the salivary bacteria are adequate biomarkers of periodontitis. Moreover, we investigated if the combination of salivary pathogens, i.e., the salivary bacterial burden, provides more diagnostic value than the levels of individual pathogens. Our final aim is to find biomarkers that can be used in developing salivary diagnostic tools. Salivary diagnostics could provide an easy assessment of periodontal risk, e.g., for health care promotion or home testing purposes.

## Materials and methods

### Participants

The study population included 462 dentate participants of the Parogene study (Buhlin et al., 2011). The original aim of the study is to identify genetic loci and variations disclosing the association between periodontitis and CAD. The Parogene study was a substudy of the Corogene study that included 5295 native symptomatic Finnish patients who were assigned to coronary angiography in Helsinki University Central Hospital, Finland, between 2006 and 2008 (Vaara et al., 2012). Ten percent of the Corogene population was randomly chosen to be invited to a comprehensive oral examination. Five hundred and eight patients participated in the clinical and radiographic oral examination at the Institute of Dentistry, University of Helsinki. The study was approved by the Helsinki University Central Hospital ethics committee (#106/2007) and all subjects gave a written informed consent.

### Oral examination

The oral examination was performed by two calibrated periodontists (Buhlin et al., 2011). Probing pocket depths (PPD) were measured from six sites of each tooth with a manual periodontal probe and BOP was registered from four sites of each tooth. The amount of alveolar bone loss (ABL) was calculated from digital panoramic radiographs by choosing the tooth with most severe attachment loss from each dentate sextant and graded into four categories by calculating the mean of the sextants: no ABL; mild, ABL in cervical third of the root; moderate, ABL in the middle third of the root; and from severe to total loss, ABL from the apical third of the root to total ABL. The study subjects completed a questionnaire on their smoking habits, recent intake of antibiotics, and previous periodontal treatment.

The dentate subjects were divided into two groups based on their periodontal status: 338 subjects (73.2%) with no periodontitis or mild periodontitis (no ABL or mild ABL or less than four sites with PPD of ≥4 mm) and 124 subjects (26.8%) with moderate to severe periodontitis (patients with ABL from moderate to severe and at least four sites with PPD of ≥4 mm) (Salminen et al., 2014).

### Saliva samples

Before the oral examination, the subjects chewed a piece of paraffin for 5 min and a minimum of 2 ml of stimulated saliva was collected. The concentrations of bacteria were analyzed from saliva samples of 492 subjects. Thirty edentulous patients were excluded from further statistical analyses.

### Quantitative PCR

Quantitative real-time PCR (qPCR) assays for *P. gingivalis, T. forsythia, P. intermedia*, and *A. actinomycetemcomitans* were performed for the saliva samples in an earlier study (Hyvärinen et al., 2012). Briefly, total bacterial DNA was isolated from the pellets derived from 500 μl of saliva and reference strain cultures using a ZR Fungal/Bacterial DNA Kit™ (Zymo Research) according to the manufacturer's instructions. The DNA concentrations were analyzed using NanoDrop 1000 spectrofotometer (Thermo Fisher Scientific). The target gene for primers (Thermo Fisher Scientific) and TaqMan probes (DNA Technology A/S) was Kdo transferase waaA and amplifications were conducted in duplicate 25 μl reactions by using Brilliant QPCR Master Mix (Agilent Technologies Inc.) and optimized concentrations of primer/probe sets (Hyvärinen et al., 2009). The thermocycling protocol used in Mx3005P Real-Time QPCR System (Agilent Technologies Inc.) was as follows: 15 min at 95°C, followed by 40 cycles of 15 s at 95°C and 1 min at 60°C. The results were analyzed using Mx3005P Real-Time QPCR System software and the bacterial concentrations were calculated from standard curves derived from serial dilutions (1 × 10^−5^–1 ng) of reference strains. The masses of bacterial genomes were calculated from genomic size data and the final results were expressed as genomic equivalents (GE)/ml saliva (Hyvärinen et al., 2009).

### Statistical analysis

The statistical analysis was performed with IBM SPSS Statistics software (version 22.0; IBM, New York, USA).

The differences of characteristics, periodontal parameters, presence of the bacteria, and salivary concentrations of the bacteria between subjects with no to mild periodontitis and subjects with moderate to severe periodontitis were analyzed by the Pearson Chi-Squared test (for categorical variables), *t*-test (for continuous, normally distributed variables: age), and the Mann-Whitney test (for continuous, non-normally distributed variables: bacterial concentrations and the number of teeth and implants).

In addition to the main periodontal diagnosis (no to mild periodontitis vs. moderate to severe periodontitis), the subjects were divided into subgroups defined by different clinical and radiographic parameters of periodontitis (the number of periodontal pockets with PPD of 4–5 mm, the number of pockets with PPD ≥ 6 mm, the percentage of BOP, and the degree of ABL). The concentrations of the bacteria were analyzed in these subgroups. Concentrations of salivary bacteria were expressed as medians with interquartile range as they were not normally distributed, and the comparisons between the subgroups of study subjects defined by different periodontal characteristics were made with the Jonckheere-Terpstra test.

The concentrations of each salivary bacteria were divided into three levels/scores as follows: low level, score 1: 0 GE/ml (concentration below the detection limit); medium level, score 2: concentration above the detection limit but below the median detectable concentration; and high level, score 3: concentration above the median detectable concentration.

The number of subjects with low, medium, or high levels of each pathogen were calculated in the groups divided according to periodontal diagnosis and in the subgroups divided according to periodontal parameters. The comparisons between the groups were made with the Chi-Squared test.

To obtain a bacterial burden index reflecting the total bacterial load in saliva for each individual, the scores of the four bacteria were summed up resulting in a number between 4 (if the concentrations of all four bacteria were below the detection limit) and 12 (if the level of each pathogen was high). After calculating the bacterial scores together, the sums were divided into tertiles, resulting in a bacterial burden index value of I (low burden), II (medium burden), or III (high burden) for each individual. The idea of a cumulative approach in salivary diagnostics has been described earlier in Gursoy et al. (2011), where the concentrations of MMP-8, IL-1β, and *P. gingivalis* were used to obtain a cumulative risk score. In the present study, we used the concentrations of the four salivary bacteria instead.

Logistic regression models were used to analyze the association of salivary bacterial concentrations and their combination as three levels and as continuous variables with periodontal diagnosis (no to mild periodontitis vs moderate to severe periodontitis) and with individual parameters of periodontitis. The models were adjusted for the number of teeth and implants (continuous variable), age (continuous variable), gender (male/female), diabetes (no/yes), and smoking (never/former/current). In further models, CAD diagnosis was added as a covariate to ensure that the heart disease status of the patients did not have an effect on the results. For the regression analyses with continuous variables, the concentrations of the bacteria were log-transformed (10-based logarithm). If the concentration was below the detection limit, the value of logarithm was set to 0. For the combinations of bacteria as continuous variables, the log-transformed bacterial concentrations were summed up.

## Results

In the whole study population, 55.2, 65.7, 24.1, and 10.8% of the subjects were positive for saliva *P. gingivalis, T. forsythia, P. intermedia*, and *A. actinomycetemcomitans*, respectively.

The study subjects were divided into two groups based on their periodontal diagnosis: no to mild periodontitis and moderate to severe periodontitis. The characteristics, periodontal parameters, the presence of the bacteria in saliva, and the salivary concentrations of the bacteria in these two groups are shown in Table 1. The subjects with moderate to severe periodontitis were older, they were more frequently current or former smokers and male, they had received periodontal treatment more often, and they had fewer teeth than those with no or mild periodontitis. They were also more frequently positive for salivary *P. gingivalis*, but not for other bacteria. Median salivary concentrations of *P. gingivalis, T. forsythia*, and *P. intermedia*, as well as the sum of the concentrations of the four bacteria, were higher in subjects with moderate to severe periodontitis compared to subjects with no to mild periodontitis (Table 1). Salivary *A. actinomycetemcomitans* concentrations did not differ significantly between the two groups (Table 1).

**Table 1:** **Characteristics of the study subjects**.

			**No—mild periodontitis (*n* = 340)**	**Moderate—severe periodontitis (*n* = 124)**	
			**N (%)**		***p*^d^**
Positive for saliva	Pg		177 (52.1)	79 (63.7)	**0.03**
	Tf		215 (63.6)	90 (72.6)	0.07
	Pi		75 (22.1)	37 (29.8)	0.08
	Aa		35 (10.3)	15 (12.1)	0.58
	*n* of species	0	81 (23.8)	24 (19.4)	0.07
		1	90 (26.5)	22 (17.7)	
		2	104 (30.6)	42 (33.9)	
		3	56 (16.5)	29 (23.4)	
		4	9 (2.6)	7 (5.6)	
Gender (men)			210 (61.8)	92 (74.2)	**0.01**
Smokers	Current		27 (7.9)	28 (22.6)	**<0.001**
	Former		127 (37.4)	59 (47.6)	**<0.001**
Diabetes			69 (20.7)	36 (29.0)	0.06
Antibiotic treatment^a^			136 (41.3)	46 (38.7)	0.66
Periodontal treatment^b^			33 (10.3)	22 (20.4)	**0.01**
			**MEDIAN (IQR)**		***p***^e^
Salivary concentration (GE/ml)^c^	Pg		679 (21,300)	31,500 (311,000)	**<0.001**
	Tf		33,100 (297,000)	265,000 (1,280,000)	**<0.001**
	Pi		4040 (70,900)	55,900 (824,000)	**<0.01**
	Aa		1100 (5780)	539 (3510)	0.43
	Sum of bacteria		38,900 (372,000)	518,000 (1,950,000)	**<0.001**
Number of teeth and implants			25.0 (7)	21.0 (12)	**<0.001**
			**MEAN (SD)**		***p***^f^
Age			62.1 (9.6)	65.3 (7.5)	**<0.001**

a Self-reported intake of antibiotics during past 6 months;

b Self-reported periodontal treatment in the past;

c In pathogen-positive subjects;

d Chi-squared test;

e Mann-Whitney test;

f t-test; Significant p-values are presented in bold face.

In addition to the main periodontal diagnosis, the subjects were divided into subgroups defined by different clinical and radiographic parameters of periodontitis. The median concentrations of salivary bacteria in these subgroups are presented in Table 2, and the number of subjects with low, medium, or high levels of each bacterium in these subgroups are presented in Supplementary Table 1. The median concentrations of *A. actinomycetemcomitans* did not differ between any of the subgroups (Table 2). The median *P. intermedia* concentrations differed between subgroups divided according to the number of sites with PPD ≥ 6 mm or the degree of ABL. The median concentrations of *T. forsythia* differed additionally between the subgroups divided based on the number of sites with PPD of 4–5 mm. The median concentrations of *P. gingivalis* differed between subgroups defined by all periodontal parameters investigated (Table 2).

**Table 2:** **Median concentrations of salivary pathogens in subgroups of pathogen-positive subjects divided according to periodontal parameters**.

	**Concentration, median (IQR)**			
	**Pg (GE/ml)**	**Tf (GE/ml)**	**Pi (GE/ml)**	**Aa (GE/ml)**
**NUMBER OF SITES WITH PPD 4–5 mm**				
0	425 (1390)	19,600 (198,000)	681 (47,600)	320 (-)
1–6	338 (25,800)	26,700 (196,000)	8115 (57,000)	2330 (41,700)
7–16	3640 (83,500)	100,000 (960,000)	6360 (144,000)	2280 (14,200)
17–	12,000 (222,000)	103,000 (652,000)	42,300 (697,000)	539 (1600)
	***p* < 0.001**	***p* = 0.01**	*p* = 0.20	*p* = 0.25
**NUMBER OF SITES WITH PPD ≥ 6 mm**				
0	499 (7710)	25,700 (309,000)	4250 (38,000)	939 (8820)
1–3	12,000 (60,100)	107,289 (852,000)	7830 (340,000)	627 (1950)
4–6	48,200 (931,000)	60,745 (329,000)	180,000 (836,000)	3710 (11,100)
7–	32,000 (364,000)	284,000 (1,570,000)	53,500 (833,000)	808 (2580)
	***p* < 0.001**	***p* < 0.001**	***p* < 0.01**	*p* = 0.70
**BLEEDING ON PROBING, %, TERTILES**				
0–26	639 (16,900)	41,631 (303,000)	4250 (107,000)	664 (28,000)
27–44	1200 (31,000)	33,100 (738,000)	21,500 (288,000)	3170 (7980)
45–100	30,300 (317,000)	88,000 (708,000)	7140 (205,000)	404 (1540)
	***p* < 0.001**	***p* = 0.03**	*p* = 0.36	*p* = 0.31
**ALVEOLAR BONE LOSS**				
None	464 (2320)	31,400 (246,000)	5420 (21300)	519 (2300)
Mild	1240 (45,700)	39,400 (308,000)	3260 (118,000)	1420 (13,000)
Moderate	21,500 (174,000)	103,000 (952,000)	46,300 (401,000)	843 (8140)
Severe—total	291,000 (884,000)	634,000 (2,390,000)	519,000 (1,790,000)	300 (4040)
	***p* < 0.001**	***p* < 0.001**	***p* = 0.01**	*p* = 0.77
**NO. OF TEETH + IMPLANTS**				
1–10	2030 (7790)	25,300 (242,000)	55,900 (864,000)	356 (1070)
11–20	32,600 (292,000)	66,100 (1,180,000)	21,500 (533,000)	8470 (47,900)
21–25	3540 (70800)	117,000 (646,000)	5850 (225,000)	808 (8980)
26–	438 (9530)	31,400 (373,000)	4040 (45,800)	797 (1720)
	***p* < 0.001**	*p* = 0.34	***p* < 0.01**	*p* = 0.59

PPD, pocket probing depth; IQR, interquartile range.

P-values were obtained by Jonckheere-Terpstra test.

Significant p-values are presented in bold face.

The combination of the four bacteria, i.e., the bacterial burden index, was calculated for each individual. The number of subjects with the bacterial burden index I, II, and III in the subgroups divided according to periodontal parameters are presented in Supplementary Table 2.

Table 3 indicates the correlations between the salivary concentrations of the four bacteria. The concentrations of all bacterial species correlated significantly with each other. The strongest correlation was found between *P. gingivalis* and *T. forsythia*.

**Table 3:** **Correlations between salivary pathogen concentrations**.

		**Pg**	**Tf**	**Pi**	**Aa**
**Pg**	Correlation coefficient	1.00	0.50	0.31	0.17
	*p*-value	.	**< 0.001**	**< 0.001**	**<0.001**
**Tf**	Correlation coefficient		1.00	0.27	0.23
	*p*-value		.	**< 0.001**	**<0.001**
**Pi**	Correlation coefficient			1.00	0.10
	*p*-value			.	**0.03**
**Aa**	Correlation coefficient				1.00
	*p*-value				.

Spearman's correlation.

Significant p-values are presented in bold face.

**Figure 1:**
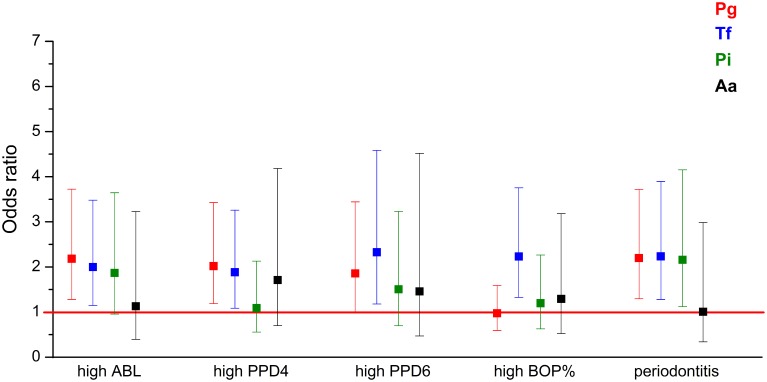
**The associations (OR) between periodontal parameters and the four bacteria investigated**. ORs are calculated for the association between the periodontal parameters and highest levels of periodontal pathogen concentrations. The regression models were adjusted for the number of teeth and implants, age, gender, smoking, and diabetes. Error bars represent 95% confidence intervals. High ABL: ABL from moderate to severe compared to no to mild ABL; high PPD4: ≥17 sites with PPD 4–5 mm compared to < 17 sites; high PPD6: ≥7 sites with PPD ≥ 6 mm compared to < 7 sites; high BOP: BOP% ≥ 40 compared to < 40%, periodontitis: moderate to severe periodontitis compared to no to mild periodontitis. ABL, alveolar bone loss; PPD, pocket probing depth; BOP, bleeding on probing.

Logistic regression models adjusted for age, gender, smoking, diabetes, and the number of teeth plus implants were used to evaluate the associations between salivary pathogen levels and periodontal parameters. Table 4 and Figure 1 present the odds ratios (OR) and 95% confidence intervals (95% CI) for the associations between periodontal parameters and salivary pathogen levels in the study population. High salivary concentrations of *P. gingivalis, T. forsythia*, and *P. intermedia* were significantly associated with moderate to severe periodontitis. When looking at individual parameters of periodontitis, high levels of *P. gingivalis* and *T. forsythia* were significantly associated with the number of 4–5 mm periodontal pockets, ≥6 mm pockets, and ABL (Table 4 and Figure 1). High level of *T. forsythia* was also associated with BOP. When the models were further adjusted for CAD status, the ORs remained similar.

**Table 4:** **The associations (OR) between periodontal parameters and the four salivary pathogens**.

**Dependent parameter**	**Bacterial concentration**	**Pg OR (95% CI)**	***p***	**Tf OR (95% CI)**	***p***	**Pi OR (95% CI)**	***p***	**Aa OR (95% CI)**	***p***
High ABL (moderate-total) reference: none-mild ABL	Low^a^	1		1		1		1	
	Medium^a^	0.99 (0.56–1.77)	0.98	1.00 (0.56–1.78)	0.99	0.90 (0.43–1.85)	0.77	2.30 (0.91–5.83)	0.08
	High^a^	2.19 (1.29–3.73)	**<0.01**	2.01 (1.15–3.49)	**0.01**	1.88 (0.96–3.65)	0.06	1.13 (0.39–3.23)	0.82
	Continuous^b^	1.19 (1.07–1.32)	**<0.001**	1.14 (1.04–1.26)	**<0.01**	1.10 (0.98–1.24)	0.12	1.14 (0.92–1.41)	0.25
High PPD4 (≥17 sites with PPD 4–5 mm) reference: < 17 sites with PPD 4–5 mm	Low	1		1		1		1	
	Medium	0.89 (0.52–1.55)	0.69	1.62 (0.93–2.81)	0.09	0.82 (0.42–1.60)	0.55	4.14 (1.69–10.2)	**0.01**
	High	2.03 (1.20–3.43)	**<0.01**	1.89 (1.10–3.27)	**0.02**	1.10 (0.57–2.14)	0.78	1.71 (0.70–4.18)	0.24
	Continuous	1.17 (1.06–1.30)	**<0.01**	1.12 (1.03–1.23)	**0.01**	1.03 (0.92–1.16)	0.62	1.25 (1.02–1.52)	**0.03**
High PPD6 (≥7 sites with PPD ≥ 6 mm) reference: < 7 sites with PPD ≥ 6 mm	Low	1		1		1		1	
	Medium	0.59 (0.27–1.28)	0.18	1.11 (0.53–2.34)	0.78	0.94 (0.40–2.25)	0.90	1.64 (0.57–4.67)	0.36
	High	1.86 (1.01–3.45)	**0.05**	2.33 (1.19–4.59)	**0.02**	1.51 (0.71–3.24)	0.29	1.46 (0.47–4.52)	0.51
	Continuous	1.14 (1.01–1.29)	**0.04**	1.19 (1.05–1.34)	**<0.01**	1.07 (0.94–1.23)	0.30	1.12 (0.88–1.43)	0.35
High BOP (highest tertile) reference: tertiles 1–2	Low	1		1		1		1	
	Medium	0.25 (0.14–0.46)	**<0.001**	1.46 (0.86–2.47)	0.16	1.65 (0.89–3.06)	0.11	1.80 (0.75–4.31)	0.19
	High	0.98 (0.60–1.60)	0.94	2.24 (1.34–3.76)	**<0.01**	1.21 (0.64–2.27)	0.56	1.29 (0.52–3.18)	0.58
	Continuous	0.98 (0.89–1.09)	0.74	1.16 (1.06–1.26)	**<0.001**	1.07 (0.96–1.19)	0.24	1.09 (0.89–1.32)	0.41
Moderate—severe periodontitis reference: no—mild periodontitis	Low	1		1		1		1	
	Medium	0.77 (0.42–1.41)	0.39	0.99 (0.55–1.78)	0.98	1.02 (0.50–2.10)	0.96	2.22 (0.89–5.51)	0.09
	High	2.20 (1.30–3.72)	**<0.01**	2.24 (1.30–3.90)	**<0.01**	2.17 (1.13–4.16)	**0.02**	1.01 (0.34–2.98)	0.99
	Continuous	1.19 (1.08–1.32)	**<0.001**	1.16 (1.06–1.28)	**<0.01**	1.14 (1.02–1.28)	**0.02**	1.11 (0.90–1.39)	0.34

a Low, below the detection limit; Medium, above the detection limit but below the median concentration; High, above the median concentration.

b The continuous analyses are expressed as OR / log-transformed GE/ml.

Models were adjusted for age, gender, diabetes, smoking, and number of teeth plus implants.

ABL, alveolar bone loss; PPD, pocket probing depth; BOP, bleeding on probing; OR, odds ratio; CI, confidence interval.

Significant p-values are presented in bold face.

**Figure 2:**
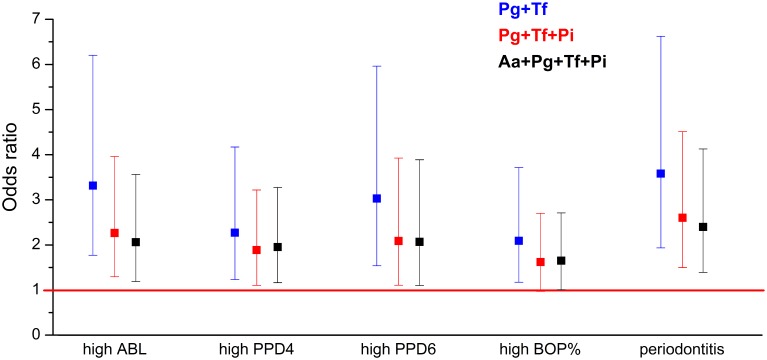
**The associations (OR) between periodontal parameters and bacterial burden index**. ORs are calculated for the association between the periodontal parameters and bacterial burden index III calculated for different bacterial combinations. The OR was calculated for the following combinations of bacteria: *P. gingivalis, T. forsythia, P. intermedia*, and *A. actinomycetemcomitans*; for *P. gingivalis, T. forsythia*, and *P. intermedia*; and for *P. gingivalis* and *T. forsythia*. ABL, alveolar bone loss; PPD, pocket probing depth; BOP, bleeding on probing.

In the adjusted regression models, the bacterial burden index reflecting the total bacterial load was associated with moderate to severe periodontitis with an OR of 2.40 (95% CI 1.39–4.13) (Table 5 and Figure 2). When *A. actinomycetemcomitans* was excluded from the combination of the bacteria, the OR was improved to 2.61 (95% CI 1.51–4.52). When *P. intermedia* was further excluded from the combination, and only the concentrations of *P. gingivalis* and *T. forsythia* were used, the OR was increased to 3.59 (95% CI 1.94–6.63) (Table 5 and Figure 2). The bacteria to be excluded one-by-one from the model were chosen because they had the weakest association with periodontitis.

**Table 5:** **The associations (OR) between periodontal parameters and bacterial burden index calculated for different bacterial combinations**.

**Dependent parameter**	**Bacterial burden index**	**With Pg, Tf, Pi, and Aa OR (95% CI)**	***p***	**With Pg, Tf, and Pi OR (95% CI)**	***p***	**With Pg and Tf OR (95% CI)**	***p***
High ABL (moderate-total) reference: none-mild ABL	I	1		1		1	
	II	1.06 (0.61–1.85)	0.84	0.97 (0.56–1.67)	0.90	1.05 (0.63–1.76)	0.85
	III	2.06 (1.19–3.56)	**0.01**	2.27 (1.30–3.97)	**<0.01**	3.32 (1.78–6.21)	**<0.01**
	Continuous^a^	1.08 (1.03–1.12)	**<0.001**	1.08 (1.03–1.13)	**<0.001**	1.11 (1.04–1.17)	**<0.001**
High PPD4 (≥17 sites with PPD 4–5 mm) reference: < 17 sites with PPD 4–5 mm	I	1		1		1	
	II	0.76 (0.44–1.33)	0.34	0.73 (0.43–1.26)	0.26	0.96 (0.58–1.57)	0.87
	III	1.95 (1.16–3.28)	**0.01**	1.90 (1.11–3.23)	**0.02**	2.28 (1.25–4.18)	**<0.01**
	Continuous	1.06 (1.02–1.11)	**<0.01**	1.06 (1.02–1.11)	**<0.01**	1.10 (1.03–1.16)	**<0.01**
High PPD6 (≥7 sites with PPD ≥ 6 mm) reference: < 7 sites with PPD ≥ 6 mm	I	1		1		1	
	II	0.85 (0.41–1.76)	0.66	0.70 (0.34–1.44)	0.33	0.76 (0.38–1.49)	0.42
	III	2.07 (1.10–3.89)	**0.02**	2.10 (1.12–3.93)	**0.02**	3.04 (1.55–5.97)	**<0.01**
	Continuous	1.07 (1.02–1.13)	**<0.01**	1.08 (1.02–1.14)	**<0.01**	1.11 (1.03–1.19)	**<0.01**
High BOP (highest tertile) reference: tertiles 1–2	I	1		1		1	
	II	0.93 (0.56–1.55)	0.78	0.98 (0.60–1.61)	0.93	0.78 (0.49–1.26)	0.31
	III	1.65 (1.01–2.71)	**0.05**	1.63 (0.98–2.71)	0.06	2.10 (1.18–3.73)	**0.01**
	Continuous	1.04 (1.00–1.08)	0.05	1.04 (1.00–1.09)	0.06	1.05 (1.00–1.11)	0.06
Moderate—severe periodontitis reference: no—mild periodontitis	I	1		1		1	
	II	0.90 (0.51–1.60)	0.72	0.81 (0.46–1.43)	0.46	0.92 (0.54–1.56)	0.76
	III	2.40 (1.39–4.13)	**<0.01**	2.61 (1.51–4.52)	**<0.01**	3.59 (1.94–6.63)	**<0.001**
	Continuous	1.09 (1.04–1.13)	**<0.001**	1.09 (1.04–1.14)	**<0.001**	1.12 (1.05–1.19)	**<0.001**

a Calculated by summing up the log-transformed concentrations of the bacteria.

Models were adjusted for age, gender, diabetes, smoking, and number of teeth plus implants.

ABL, alveolar bone loss; PPD, pocket probing depth; BOP, bleeding on probing; OR, odds ratio; CI, confidence interval.

Significant p-values are presented in bold face.

Since the combination of *P. gingivalis* and *T. forsythia* was most strongly associated with periodontitis, we investigated this combination further using the modified bacterial burden index calculated based on these two species. Table 6 shows the number of individuals with no to mild periodontitis and with moderate to severe periodontitis having the modified bacterial burden index of I, II, or III. Half of those with no to mild periodontitis had an index value of I. However, also more than one third of individuals with moderate to severe periodontitis had an index value of I. The sensitivity of the bacterial burden index III for detecting periodontitis was 31% and the specificity was 89%.

**Table 6:** **Distribution of patients according to their periodontal diagnosis in the classes of the bacterial burden index calculated using the combination of salivary *P. gingivalis* and *T. forsythia* concentrations**.

	**No—mild periodontitis N (%)**	**Moderate—severe periodontitis N (%)**
Bacterial burden index I	169 (50%)	47 (38%)
Bacterial burden index II	133 (39%)	38 (31%)
Bacterial burden index III	36 (11%)	39 (31%)
Total count	338 (100%)	124 (100%)

## Discussion

Our results show that salivary concentrations of *P. gingivalis, T. forsythia*, and *P. intermedia* are associated with periodontitis. The combination of salivary *P. gingivalis* and *T. forsythia* had the strongest association with periodontitis when compared to the four pathogens analyzed individually and in combinations. The results were independent of age, gender, smoking, presence of diabetes, or CAD, as well as of the number of teeth and implants.

There is a limited number of previous studies that utilize salivary bacterial concentrations measured by qPCR in diagnostics of periodontitis. Saygun et al. showed in a sample of 150 systemically healthy subjects that *P. gingivalis, T. forsythia*, and *P. intermedia* occurred with significantly higher copy numbers in the saliva of patients with gingivitis, chronic periodontitis, and aggressive periodontitis when compared to periodontally healthy individuals (Saygun et al., 2011). Similarly, Hyvärinen et al. found that salivary levels of *P. gingivalis, T. forsythia*, and *P. intermedia* were higher in subjects with periodontitis (*n* = 84) compared to periodontally healthy controls (*n* = 81) (Hyvärinen et al., 2009). Sawamoto et al. detected higher *P. gingivalis* and *T. forsythia* concentrations in the saliva of periodontitis patients (*n* = 29) compared to healthy subjects (*n* = 20) (Sawamoto et al., 2005), and He et al. found higher concentrations of *P. gingivalis* and *P. intermedia* in patients with chronic periodontitis (*n* = 25) compared to controls (*n* = 60) (He et al., 2012). The results of these previous reports are concordant with our results. However, the sample sizes have been relatively small and the study design has been case-control.

Since the bacterial burden index calculated using *P. gingivalis* and *T. forsythia* was most strongly associated with periodontitis, we investigated this combination further. Half of the patients with no to mild periodontitis had bacterial burden index value of I representing the lowest pathogen burden. However, also more than one third of individuals with moderate to severe periodontitis had index value of I. This suggests that the levels of the salivary pathogens analyzed are likely to be low in periodontally healthy subjects, but they might be low also in individuals with periodontitis. Since periodontitis results from a dysbiotic state of oral microbiota, a large number of pathogenic species are involved. Possibly analyzing a higher number of species among the 1000 found in the oral cavity (Wade, 2013) would increase the sensitivity of the burden index. Furthermore, the salivary bacterial composition might not always directly reflect the bacteria in plaque biofilm. In Yamanaka et al. (2012), bacterial populations of saliva and supragingival plaque were analyzed before and after periodontal treatment. Following periodontal therapy, microbial richness and biodiversity were significantly decreased in the plaque microbiota, but not in saliva. These results suggest that the contribution of plaque microbes to salivary bacterial composition is limited. It is possible that saliva reflects better other oral bacterial communities than dental plaque, such as the mucosal microbiota.

In a previous study, *A. actinomycetemcomitans* showed higher salivary copy numbers only in subjects with aggressive periodontitis, but not in subjects with chronic periodontitis (Saygun et al., 2011). In Hyvärinen et al. (2009), the presence of *A. actinomycetemcomitans* was more common in periodontitis patients, but the salivary concentrations did not differ between periodontitis cases and controls. Also in the present study, high level of *A. actinomycetemcomitans* was not associated with any of the periodontal parameters investigated. This may be due to the serological heterogeneity of *A. actinomycetemcomitans*. The serotype b is considered to be etiologically linked to certain types of periodontal disease (Zambon et al., 1983), while the role of other serotypes is not that clear. The assay used in the present study was carefully designed to recognize all serotypes of the species (Hyvärinen et al., 2009), but it may be useful to combine it with serotype detection. *P. intermedia* was not associated with any of the individual parameters either, but it was associated with moderate—severe periodontitis.

In the study of Saygun et al., the diagnostic sensitivity for periodontitis was 89.2 for *P. gingivalis* and *T. forsythia* and 86.5 for *P. intermedia*, with specificities ranging from 83.8 to 94.6 (Saygun et al., 2011). The sensitivities are considerably higher than in our study, but the specificities are at a comparable level. In addition to methodological differences, this may result from the different study designs. Their study had a case-control setting which included only individuals with teeth without any deepened periodontal pockets or attachment loss, and patients with at least nine posterior teeth with 5–7 mm pocket depth, i.e., patients with moderate to severe periodontitis. No “borderline” cases or cases with mild periodontitis were included, even though they are likely to complicate the diagnostics in reality. In our study, the subjects were randomly selected from the Corogene study cohort, which included all patients assigned to coronary angiography in Helsinki University Central Hospital under a certain time period. The selection was not based on the periodontal status of the individuals and they represented all stages of periodontal conditions. Study subjects were middle-aged or older, and thus belonging to the age group that is most often affected by chronic periodontitis.

Systemic diseases may affect the composition of saliva, thereby posing challenges to salivary diagnostics. For example, the concentrations of salivary interleukins and matrix metalloproteinases may be influenced by smoking, diabetes, tumors, or joint diseases (Costa et al., 2010; Rathnayake et al., 2013a,b). In the present study, all study subjects were assigned to coronary angiography because of cardiologic problems. 25% of the patients also had diabetes, which was taken into account in the analyses. These systemic diseases or smoking did not seem to confound the diagnostics of periodontitis based on salivary pathogens.

A good diagnostic marker of periodontitis should be applicable in entire populations regardless of systemic diseases, the number of teeth, or smoking habits of the individuals. Two previous studies indicated that a combination of salivary biomarkers and subgingival plaque pathogens is associated with periodontitis and its progression more strongly than individual markers (Ramseier et al., 2009; Kinney et al., 2011). Moreover, the combination of the levels of three selected salivary biomarkers, namely MMP-8, IL-1β, and *P. gingivalis*, was associated with periodontitis better than any of the markers alone (Gursoy et al., 2011; Salminen et al., 2014). The OR for the association between periodontitis and the combination of *P. gingivalis*, IL-1β, and MMP-8 in the Parogene population was 6.13 (95% CI 3.11–12.09) compared to 3.59 (1.94–6.63) in the present study for the combination of *P. gingivalis* and *T. forsythia*, which suggests that analyzing host-derived salivary biomarkers in addition to periodontal pathogens would be beneficial to periodontal diagnostics (Salminen et al., 2014).

Hyvärinen et al. observed that the combination of salivary *P. gingivalis, P. intermedia, and A. actinomycetemcomitans* had the highest area under the ROC curve when different pathogen combinations were analyzed for diagnostics of periodontitis (Hyvärinen et al., 2009). However, the sample size of the study was limited and it was a case-control study including only subjects with healthy periodontium or substantial periodontitis, which might cause discrepancy in the results.

In our study, the highest OR for the presence of moderate—severe periodontitis was achieved by combining salivary *P. gingivalis* and *T. forsythia* concentrations. As these pathogens belong to the “red complex,” this finding strengthens their importance in periodontitis. The cumulative strategy appears to be useful in the analysis of salivary bacteria as biomarkers of periodontitis.

## Funding

This work was supported by the Finnish Dental Society Apollonia, the Helsinki Doctoral School in Health Sciences, the Sigrid Juselius Foundation (grant to PP), and the Academy of Finland (grant to PP #1266053).

### Conflict of interest statement

The authors declare that the research was conducted in the absence of any commercial or financial relationships that could be construed as a potential conflict of interest.
